# Phytochemical Profile, Antioxidant and Cytotoxic Potential of *Capsicum annuum* (L.) Dry Hydro-Ethanolic Extract

**DOI:** 10.3390/pharmaceutics16020245

**Published:** 2024-02-07

**Authors:** Ionuț Mădălin Ivan, Violeta Popovici, Carmen Lidia Chițescu, Liliana Popescu, Emanuela Alice Luță, Elena Iuliana Ilie, Lorelei Irina Brașoveanu, Camelia Mia Hotnog, Octavian Tudorel Olaru, George Mihai Nițulescu, Rica Boscencu, Cerasela Elena Gîrd

**Affiliations:** 1 Faculty of Pharmacy, University of Medicine and Pharmacy “Carol Davila”, Traian Vuia 6, 020956 Bucharest, Romania; ionut.ivan@drd.umfcd.ro (I.M.I.); liliana.popescu22@umfcd.ro (L.P.); emanuela.luta@umfcd.ro (E.A.L.); elena.ionita@drd.umfcd.ro (E.I.I.); george.nitulescu@umfcd.ro (G.M.N.); rica.boscencu@umfcd.ro (R.B.); cerasela.gird@umfcd.ro (C.E.G.); 2 “Costin C. Kiriţescu” National Institute of Economic Research—Center for Mountain Economics (INCE-CEMONT), Romanian Academy, 725700 Vatra-Dornei, Romania; 3 Faculty of Medicine and Pharmacy, “Dunărea de Jos” University of Galați, A.I. Cuza 35, 800010 Galați, Romania; carmen.chitescu@ugal.ro; 4 Center of Immunology, “Stefan S. Nicolau” Institute of Virology, Romanian Academy, 285 Mihai Bravu Ave., 030304 Bucharest, Romania; lorelei.brasoveanu@virology.ro (L.I.B.); camelia.hotnog@virology.ro (C.M.H.)

**Keywords:** *Capsicum annuum* (L.) fruits, dry hydro-ethanolic extract, UHPLC–HRMS/MS, phenolic metabolites, antioxidant activity, in vitro and in vivo cytotoxicity

## Abstract

*Capsicum annuum* (L.) is one of the essential spices most frequently used in our daily routine and has remarkable ethnobotanical and pharmacological properties. Its fruits are rich in vitamins, minerals, carotenoids, and numerous other phenolic metabolites with a well-known antioxidant activity. Regular consumption of chili fruits may have a positive influence on human health. Therefore, we investigated a commercially available chili fruit powder in the present study, extracting it with 50% ethanol. The dried hydro-ethanolic extract (CAE) was thoroughly analyzed using ultra-high-performance liquid chromatography coupled with high-resolution mass spectrometry (UHPLC–HRMS/MS), and 79 bioactive phenolic constituents were identified. Then, we quantified the main phenolic compounds and found a polyphenol content of 4.725 ± 1.361 mg Eq tannic acid/100 g extract and a flavonoid amount of 1.154 ± 0.044 mg Eq rutin/100 g extract. Phenolic secondary metabolites are known for their dual redox behavior as antioxidants/pro-oxidants, underlying their numerous benefits in health and disease. Thus, the antioxidant potential of CAE was evaluated using three methods; our results could explain the protective effects of chili fruits: IC_50_DPPH = 1.669 mg/mL, IC_50_ABTS = 0.200 mg/mL, and EC_50_FRAP = 0.561 mg/mL. The pro-oxidant potential of phenolic compounds could be a basis for CAE cytotoxicity, investigated in vitro on tumor cell lines and in vivo on *Daphnia* sp. Results demonstrated the dose- and time-dependent CAE’s cytotoxic activity; the highest antiproliferative activity was recorded on colon (LoVo) and breast (MDA-MB-231) cancer cell lines after 48 h of exposure (IC_50_ values < 200 µg/mL). In vivo testing on *Daphnia* sp. reported a potent CAE cytotoxicity after 48 h and embryonic developmental delays. Extensive data analyses support our results, showing a significant correlation between the CAE’s concentration, phenolic compound content, antioxidant activity, exposure time, and the viability rate of different tested cell lines.

## 1. Introduction

The fruit of the chili pepper (*Capsicum annuum* L., Solanaceae) is one of the most appreciated natural products worldwide. A chili-pepper-rich diet in human daily meals can be helpful in alleviating micronutrient dietary deficiency due to appreciable amounts of valuable constituents (quantified in the dried product): carbohydrates (55.33–55.96%), proteins (20.19–21.50%), lipids (7.55–9.75%), dietary fibers (35.05–37.07%), vitamin C (1.36–2.02%), and minerals (potassium, phosphorus, magnesium, calcium, iron, natrium, copper, and zinc). The presence of phytochemicals with antioxidant properties in chili pepper fruit is also essential in preventing chronic diseases and underlines its extensive use in multiple cuisines. It also exhibits appetite stimulatory, anti-inflammatory, antibacterial, antifungal, antiviral, antiseptic, cytotoxic, and anticancer activities [1].

The main bioactive compounds of *C. annuum* fruits have phenolic structures belonging to different classes. The most common are polyphenols, flavonoids, and phenolic acids, with good solubility in alcohol and water. Chili fruits also contain specific phenolic constituents, capsaicinoids; they are non-volatile alkaloids with poor solubility in water and good one in polar solvents (ethanol, methanol, acetone, hexane). They are responsible for the fruits’ pungency and are currently used in treating post-herpetic neuralgia, diabetic neuropathy, psoriasis, and osteoarthritis [2].

Various solvents are used for bioactive constituents’ optimal extraction (methanol, ethanol, acetone, water or hydro-alcoholic solutions, acetonitrile, and hexane) through different protocols: maceration, Soxhlet extraction, magnetic stirring, enzymatic extraction, microwave- and ultrasound-assisted extraction, pressured liquids, and supercritical fluid extraction [3]. Recently, Kostrzewa et al. obtained carotenoids and fatty acids [2] using ethanol as a co-extractant in supercritical CO_2_ extraction. Chilkzuk et al. noted substantial differences in the chemical profile and bioactivity of chili extracts in 80% ethanol by mechanical homogenization, water, and methanol–water (40% and 70%) correlated to their lipophilicity [4]. The UPLC-ESI-QTOF analysis identified 49 phenolic constituents in the 80% ethanol extract of chili pepper; they also identified saccharides, malic acid, ascorbic acid, and carotenoids. 

Groja et al. [5] obtained a chili extract in 80% methanol through a similar method and identified 45 constituents using RP-HPLC-DAD-QTOF-MS/MS: capsaicinoids, fatty acids, sphingolipids, flavonoids, organic acids, phenolic acids, saponins, and diterpenes. Recently, in ultrasound-assisted hydro-ethanolic extract, through HPLC-ESI-TOF-MS [6], Verardo et al. identified 43 polar compounds, mainly with phenolic structures (phenolic acids, flavonoids, monoterpenes, and diterpenes).

In the present study, we aim to investigate a commercially available pre-ground and dried chili fruit powder. For this purpose, a hydro-ethanolic dry extract of *C. annuum* fruits (CAE) was obtained using a reflux extraction process in 50% ethanol, rotary evaporation, and freeze-drying. Then, we performed a complex phenolic compounds analysis for CAE using ultra-high-performance liquid chromatography coupled with high-resolution mass spectrometry (UHPLC–HRMS/MS). We also quantified the main classes of phenolic metabolites (polyphenols and flavonoids) and several representatives.

The CAE antioxidant potential was evaluated in vitro through free radical scavenging (DPPH and ABTS) and reducing power (FRAP), and its cytotoxicity was analyzed in vitro and in vivo. Several standardized human carcinoma-derived adherent cell lines of different histological origin were used: hepatocellular (HEP G2), colon (LoVo and HT-29), breast (MDA-MB-231), ovary (SK-OV-3), and tongue (PE/CA-PJ49). The antiproliferative activity of CAE was evaluated using MTS assay, with classical oncolytic drugs as the positive controls. Additionally, the CAE toxicity was assessed in vivo on two *Daphnia* species, whereas the teratogenic potential was evaluated by applying the embryo test on *Daphnia magna* embryos. 

Extensive data analyses support our results, showing a significant correlation between the CAE concentration, exposure time, phenolic compound content, antioxidant activity, and cytotoxic effects.

## 2. Materials and Methods

### 2.1. Materials

#### 2.1.1. Chemicals

All chemicals were of analytical grade. Analytical standards of 30 compounds were purchased from Sigma-Aldrich, Darmstadt, Germany. Methanol and ethyl alcohol, HPLC grade, were purchased from Merck Romania; formic acid (98%) and ultrapure water (LC-MS grade) were also purchased from Merck (Merck Romania, Bucharest, Romania). The Pierce LTQ Velos ESI positive and negative ion calibration solutions (Thermo Fisher Scientific, Karlsruhe, Germany) calibrated the Orbitrap Mass Spectrometer.

The standard phenolic compounds (8 phenolic acids, 7 isoflavones, and 15 flavonoids), ethanol, sodium acetate, AlCl_3_, DPPH, ABTS ammonium salt, trichloroacetic acid, phosphate buffer (pH = 6.6), ascorbic acid, K_3_(FeCN)_6_, and FeCl_3_ were purchased from Sigma-Aldrich, Germany. Methanol and ethanol, potassium persulfate, formic acid (98%), and ultrapure water (LC-MS grade) were provided by Merck (Merck Romania SRL, Bucharest, Romania). The Pierce LTQ Velos ESI positive and negative ion calibration solutions (Thermo Fisher Scientific, Karlsruhe, Germany) calibrated the Orbitrap Mass Spectrometer.

#### 2.1.2. C. annuum Extract Preparation

*C. annuum* (L.) fruits were purchased as Cayenne Pepper Eco Powder (Lebensbaum, Tree of Life; Ulrich Walter GmbH, Diepholz, Germany); 50 g of pepper powder was subjected to reflux extraction with 50% ethanol (Sigma-Aldrich, Darmstadt, Germany) as previously described [7]. After filtration, the obtained extract (CAE) was concentrated in a rotary evaporator (Buchi; Vacuum Pump V-700) and lyophilized (Christ Alpha 1-2/B Braun, Biotech International Ltd., Dhaka, Bangladesh). The extraction yield was 7.65%.

#### 2.1.3. Cell Lines

The cell lines used throughout the in vitro experiments were obtained from international cell banks (“American Type Culture Collection”—ATCC (Manassas, VA, USA) and “European Collection of Authenticated Cell Cultures”—ECACC (Porton Down, UK)), as follows:

- Cell lines derived from colon tumors:

(a) The LoVo cell line was initiated starting from a metastatic tumor fragment obtained from the supraclavicular region of a 56-year-old Caucasian man diagnosed with colorectal adenocarcinoma, stage IV, Dukes’ C grade. It is an adhesive cell line with epithelial morphology. It does not show mutations in TP53 (p53 w/t).

(b) The HT-29 cell line was isolated from the primary tumor of a 44-year-old Caucasian patient with colon adenocarcinoma. It is an adherent line with epithelial morphology, showing mutations in TP53.

- Cell line derived from breast tumor:

The MDA-MB-231 cell line is an adherent line isolated from the pleural fluid of a 51-year-old Caucasian patient with breast adenocarcinoma. It presents an epithelial morphology, is triple negative (for estrogen, progesterone, and HER-2 receptors), and has mutations in TP53.

- Cell line derived from ovarian tumor:

The SK-OV-3 cell line is derived from an ovarian adenocarcinoma of a 64-year-old Caucasian patient. The line is adherent, has epithelial morphology, and has a point mutation in the TP53 gene.

- Cell line derived from liver tumor:

The HEP G2 cell line is derived from a well-differentiated hepatocellular carcinoma and was isolated from the liver biopsy of a 15-year-old Caucasian man.

- OSCC cell line derived from tongue tumor:

The cell line PE/CA-PJ49 is an adherent line with epithelial morphology derived from a squamous tongue carcinoma from a 57-year-old Caucasian man.

- HUVEC endothelial cells were obtained from cells isolated from the human umbilical cord, immortalized in the laboratory, and used as normal control cells.

#### 2.1.4. Materials for In Vitro Studies

- Equipment and consumables

The following equipment was involved: inverted microscope, AURA laminar flow hood (LAF Technologies Pty Ltd., Melbourne, Australia), refrigerated centrifuge, CO_2_ humidified atmosphere incubator (Thermo Fisher Scientific, Waltham, MA, USA), and Dynex ELISA reader (Dynex Technologies—MRS, Chantilly, VA, USA).

The consumables comprised 25 cm^2^, 75 cm^2^, or 150 cm^2^ culture plates with ventilated plugs, with or without filter, 96-well flat bottom culture plates, polypropylene centrifuge tubes of 15 and 50 mL, and sterile pipettes.

- Culture media and reagents:

In the in vitro studies on cell lines, various materials were used: Dulbecco’s Modified Eagle Medium (DMEM; PAN Biotech, Aidenbach, Germany), cell-washing medium Hanks’ Balanced Buffer Solution (HBSS), 200 mM L-glutamine, fetal bovine serum (FBS), 100 mM ethylenediaminetetraacetic acid (EDTA), phosphate-buffered saline (TFS), dimethyl sulfoxide (DMSO; Sigma-Aldrich, St. Louis, MO, USA), antibiotic mixture (10,000 U/mL penicillin and 10,000 µg/mL streptomycin) (Biochrom GmbH, Berlin, Germany), Trypan Blue and CellTiter 96^®^ AQueous One Solution Cell Proliferation Assay (MTS) kit (Promega, Madison, WI, USA).

- Standard Anticancer Agents:

The chemical drugs (5-fluorouracil, cisplatin, and doxorubicin) used as positive controls for cytotoxicity assays were purchased from Sigma-Aldrich Chemie GmbH, Schnelldorf, Germany. 

### 2.2. Identification and Quantification of Phenolic Constituents by Ultra-High-Performance Liquid Chromatography Coupled with High-Resolution Mass Spectrometry (UHPLC–HRMS/MS)

The phenolic profile of CAE was established based on non-targeted tandem mass spectrometry (MS-MS) using the hyphenated technique represented by ultra-high-performance liquid chromatography (UHPLC) coupled with the Q-Exactive high-resolution mass spectrometer (HRMS). The same method was used to quantify selected phenolic compounds for each available analytical standard (Sigma-Aldrich Chemie GmbH, Schnelldorf, Germany).

The standard phenolic stock methanol solutions of 1.0 mg/mL concentration were prepared; then, a series of mixed-working standard solutions (concentration ranged from 0.05 to 1.0 µg/mL) were obtained by successive dilutions with 20% methanol. All solutions were stored at −20 °C before use. 

#### 2.2.1. LC Parameters 

The analysis platform was a Thermo Scientific Dionex Ultimate 3000 UHPLC system consisting of an RS pump coupled with a WPS-3000RS autosampler and an Accucore Column C18 (150 × 2.1 mm, 2.6 µm). A 35-min gradient and a temperature of 40 °C were applied. The mobile phase consisted of 500 µL/L formic acid in ultrapure water (pH = 2.5) and 500 µL/L formic acid in methanol. The step gradient was as follows: 0–1 min 100% A; 1.0–10.0 min linear increase to 30% B; 10.0–26.0 min linear increase to 100% B, and held for 4.0 min; 30.0–32.5 min decreasing to 0% B; equilibration time of 2.5 min. The run was executed at 0.3 mL/min for 35 min. The data were achieved using Chromeleon 7.2 Software (Thermo Fisher Scientific, Waltham, MA, USA).

#### 2.2.2. MS Parameters 

A heated electrospray ionization (HESI) ion source was used for the ionization in the negative mode [8]. The ion source parameters were optimized, the nitrogen as sheath, and the auxiliary gas flow rate was set to 8 and 6 units, respectively. The system temperature was 300 °C, the electrospray voltage was set to 2800 V, and the S-lens RF level was set to 50. 

The full-scan HRMS analysis was performed using a Q-Exactive Mass Spectrometer. Full-scan data in the negative mode were acquired at a power of 70,000 FWHM at a scan range of *m*/*z* 100–1000 Da. The automatic gain control (AGC) was set at 3 × 106, the injection time was 200 ms, and the scan rate was 2 scan/s. The calibration solution in positive and negative mode performed external calibration. A variable data independent acquisition (vDIA) approach was selected for untargeted structures from HRMS/MS analysis. 

Six scan events were combined: one whole scan event and five MS-MS events. The precursor ion ranges from *m*/*z* 95–205, 195–305, 295–405, 395–505, and 500–10,005 were consecutively selected in the MS2 scan events. They were fragmented in the HCD cell and measured in five separate Orbitrap scans at a power of 35,000 FWHM. The fragmentation was performed at a normalized collision energy of 30, 60, and 80. Moreover, an AGC of 1 × 106, an injection time of 100 ms, and a mass tolerance window of 5 ppm were selected as C-trap parameters for all scan events, and the Quan/Qual Browser Xcalibur 2.3 (Thermo Fisher Scientific, Karlsruhe, Germany) processed all the data. 

In MS-MS analysis, at least two fragment ions were detected by comparing them to the standards. The structures of the compounds without available references were presumed based on high-accuracy analysis of deprotonated precursors and fragment ions of specific components. The chemical elemental composition for each target peak was assigned within a mass error of 2 ppm using the chemical ChemSpider database (www.chemspider.com, accessed on 15 October 2023). A self-built chemical database of *C. annuum* phenolic compounds was assessed. The fragment ions from MS-MS analysis were used to further confirm the chemical structure by comparing the analysis results with MS-MS data from the NORMAN MassBank (https://massbank.eu/MassBank/, accessed on 15 October 2023), mzCloude Advanced Mass Spectral Database (https://www.mzcloud.org/, accessed on 15 October 2023), and PubChem (https://pubchem.ncbi.nlm.nih.gov/, accessed on 14 October 2023). The ACDLabs MS Fragmenter 2019.2.1 software was used to generate the fragmentation patterns of the identified compounds for comparison.

### 2.3. Total Polyphenols Content (TPC)

The Folin–Ciocalteu reagent was used following a spectrophotometric method described extensively in a previously published article [9]. The absorbances were measured at 725 nm (Jasco V-530 spectrophotometer; Tokyo, Japan), and tannic acid was the standard for the calibration curve in a linear concentration range of 2–9 µg/mL. The TPC is expressed as mg Eq tannic acid/100 g CAE.

### 2.4. Total Flavonoids (TF)

The quantification method was based on the reaction of flavonoids and AlCl_3_, detailed in a previously published article. The absorbance values were measured at 427 nm, using rutin as standard. The TF was quantified as mg Eq rutin/100 g CAE [10].

### 2.5. Antioxidant Activity 

#### 2.5.1. 2,2-Diphenyl-1-Picrylhydrazyl Free Radical Scavenging Assay (DPPH)

Under an antioxidant, the purple free radical 2,2-diphenyl-1-picrylhydrazyl (DPPH) formed its corresponding yellow hydrazine. The absorbance value was measured at λ = 515 nm. The IC_50_ value was determined from inhibition curves and their linear equations [8].

#### 2.5.2. 2,20-Azinobis-3-Ethylbenzotiazoline-6-Sulfonic Acid Assay (ABTS)

The turquoise-colored ABTS radical resulted from a potent oxidizing agent (potassium persulfate) reaction with the ammonium salt of 2,2′-azino-bis(3-ethylbenzothiazoline-6-sulfonic acid). Under the action of the antioxidant, the intensity of the color was reduced to colorless. The absorbance was determined by spectrophotometry at λ = 734 nm. The IC_50_ value was calculated from inhibition curves and their linear equations [7].

#### 2.5.3. Ferric Reducing Antioxidant Power Assay (FRAP)

The antioxidant analyte reacted with Fe^3+^ reducing to Fe^2+^, imprinting blue. The coloration intensity was directly proportional to the antioxidant activity. The absorbance values were measured at λ = 700 nm (spectrophotometer Jasco V-530), compared to the control (prepared under the same conditions without sample solution). It was expressed as an EC_50_ value; it represented the sample concentration at which the absorbance has a value of 0.5 or half the concentration at which the antioxidant activity is maximum, determined by the trendline equation [11]. 

### 2.6. In Vitro CAE-Mediated Cytotoxicity 

#### 2.6.1. Cell Cultures and Treatments

Both normal and tumor human cell lines were cultivated in 25 and 75 mm^3^ culture flasks in complete medium of DMEM/F12, 10% FBS, 2 mM L-glutamine, antibiotics were added (100 U/mL penicillin and 100 µg/mL streptomycin), and then incubated at 37 °C in a 5% CO_2_ humidified atmosphere. For routine maintenance in culture and growth, the cells were detached from flasks with a non-enzymatic solution of PBS/1 mM EDTA, washed twice in PBS, and further cultivated. For the cytotoxicity assays, cells were cultivated in 96-well flat bottom plates, and after 24 h, when cells achieved around 60% confluence, they were treated for various periods of time with different concentrations of CAE or the drugs (5-FU, CisPt, DOX), used as positive test controls. 

#### 2.6.2. Evaluation of Cellular Cytotoxicity by Colorimetric Technique (MTS)

The MTS assay investigated the potential cytotoxicity of CAE on tumor cell lines compared to normal cells. The antiproliferative effect of the bioactive compound was compared to standard anticancer drugs used as positive controls: 5-fluorouracil (5-FU), cisplatin (CisPt), and doxorubicin (DOX); the abbreviations are in accordance with https://www.allacronyms.com/ (accessed on 2 November 2023). 

The colorimetric assay used to evaluate CAE-induced cytotoxicity was the CellTiter 96^®^ AQueous One Solution Cell Proliferation Assay (MTS) kit (Promega, USA), which contains MTS tetrazole compound (3-(4,5-dimethylthiazol-2-yl)-5-(3-carboxymethoxy-phenyl)-2-(4-sulfophenyl)-2*H*-tetrazolium) and PES (phenazine ethosulfate) reagent, a cationic dye with high chemical stability. Through MTS-PES interaction, the stable complex of formazan is formed, a compound that can be spectrophotometrically quantified by reading the absorbance at the wavelength of 490 nm. The metabolically active cells can reduce the yellow tetrazolium salt MTS to colored formazan, which is soluble in cell culture medium [12].

Culture 96-well flat bottom microtiter plates (Promega) that contain cells grown in 100 µL culture medium, treated with the CAE of different concentrations for 24 and 48 h, were added by 20 µL/well of MTS reagent. Then, the plates were placed for 4 h at 37 °C in a 5% CO_2_ humidified atmosphere and mildly agitated every 15 min. The absorbance values of reduced MTS to formazan were spectrophotometrically measured at 490 nm. Data were expressed as percentages of cell viability in comparison with untreated cells, considered 100% viable, using the following equation:

(1) $$\mathrm{Viability}\;\%=100\times \frac{T-B}{U-B}$$

T = optical density of treated cells. 

B = optical density of the blank (culture medium, in the absence of cells).

U = optical density of untreated cells.

The cytotoxicity assays were realized in triplicate, and the results were expressed as mean values ± standard deviations (SD). An additional experiment was performed in the absence of cells by testing all the concentrations of CAE or drugs for their potential interference with MTS reagent; during calculations, their absorbance values were extracted.

### 2.7. The 48 Hours Acute Toxicity Test Using Daphnia magna and Daphnia Pulex 

#### 2.7.1. Principle of the Method

Larval daphnids, placed in individual containers, are exposed to various concentrations of test samples for 48 h. Mortality is the endpoint of this assay: https://clu-in.org/download/ert/2024-r00.pdf (accessed on 8 December 2023).

#### 2.7.2. Technique

The daphnids belonging to species *Daphnia magna* and *Daphnia pulex* were chosen based on their size from parthenogenetic cultures maintained in an artificial medium for 24 h before testing [13]. The assay was performed in 24-well culture plates (Greiner Bio-One), each well containing around 10 organisms. A concentration of 1% DMSO served as a negative control, and capsaicin as a positive control. Six different CAE concentrations ranging from 31.25 to 1000 μg/mL were tested. In contrast, capsaicin was first tested at concentrations between 7.5 and 62.5 µg/mL, and following the pre-screening results, the range was set between 0.2 and 6.25 μg/mL. Each sample was duplicated, and the lethality was monitored at 24 and 48 h. The 50% lethal concentrations (LC_50_) and the 95% confidence interval (CI95%) of LC_50_ values were determined using the least square fit method (GraphPad Prism v 5.1 software).

### 2.8. Daphnia magna Embryonic Development Assay

Following the results obtained in the previous test, CAE at 31.5 µg/mL and capsaicin at 6.25 µg/mL were tested. Two replicates were assessed for each sample, and the results were compared with untreated control. The embryo test was performed according to the protocol of Wang et al. [14] with some modifications. Briefly, the embryos were obtained from female daphnids and incubated with the sample concentrations in the dark at constant temperature and humidity (25 °C, 75% RH). Every 24 h, the embryos were subjected to microscopic examination (bScope^®^ microscope, Euromex Microscopen BV, Arnhem, The Netherlands) to identify the developmental stages and abnormalities.

### 2.9. Data Analysis

The statistically significant differences (*p* < 0.05) between various experimental groups were established using the one-way ANOVA test from Microsoft 365 Excel^®^ v.2023 (Microsoft Corporation, Redmond, WA, USA), Levene’s test, Fisher’s F-test, Bartlett’s test, and *t*-test for two independent samples from XLSTAT 2023.1.4. by Lumivero (Denver, CO, USA) [15].

The correlations between the bioactive constituents of the extracts and their antioxidant activity and cytotoxicity were determined using principal component analysis performed with XLSTAT 2023.1.4. by Lumivero (Denver, CO, USA) through Pearson correlation. The level of probability value *p* < 0.05 indicates statistically significant differences. 

## 3. Results

### 3.1. Identification of Bioactive Compounds by UHPLC–MS

**Figure 1:**
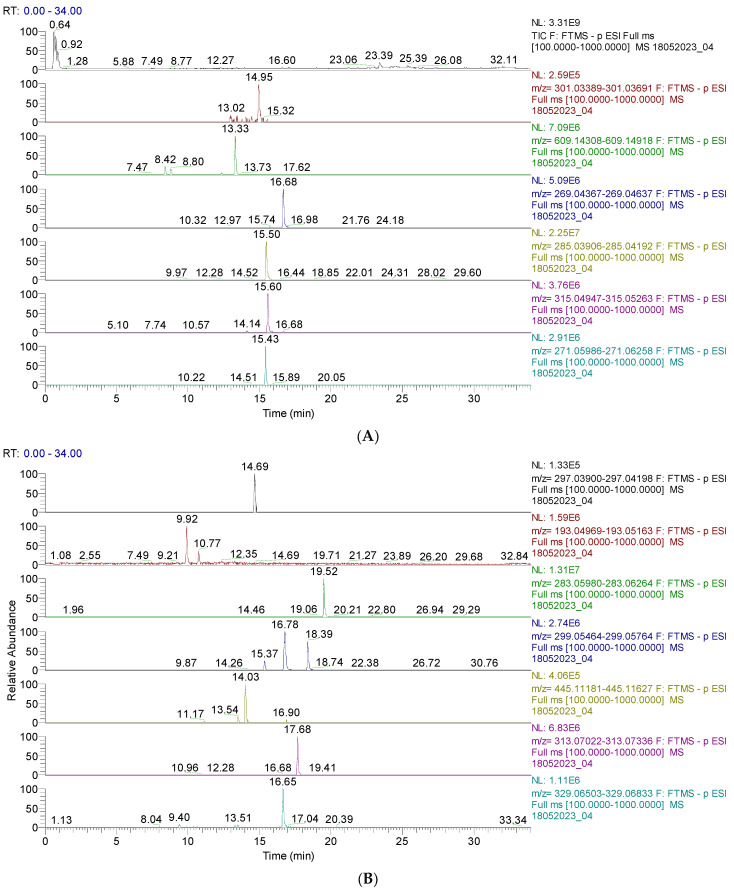
(**A**). UHPLC–HRMS/MS chromatogram of flavonoids identified in CAE; from top to bottom: quercetin (*m*/*z* = 301.035, Rt = 14.95); rutin (*m*/*z* = 601.946, Rt = 13.33); apigenin (*m*/*z* = 269.045, Rt=16.65); baptigenin (*m*/*z* = 285.040, Rt = 15.50); 6-methoxyluteolin (*m*/*z* = 315.051, Rt = 16.69); naringenin (*m*/*z* = 271.061, Rt = 15.43). (**B**). UHPLC–HRMS/MS chromatogram of isoflavones and phenolic acids identified in CAE; from top to bottom: irilone (*m*/*z* = 297.040, Rt = 14.69); ferulic acid (*m*/*z* = 193.050, Rt = 9.92); biochanin A (*m*/*z* = 283.061, Rt = 19.52); pratensein (*m*/*z* = 299.056, Rt = 16.78); chrysoeriol (*m*/*z* = 299.056, Rt = 18.39); sissotrin (*m*/*z* = 445.014, Rt = 14.03); irisolidone (*m*/*z* = 313.071, Rt = 17.68); tricin (*m*/*z* = 329.066, Rt = 16.65).

**Figure 2:**
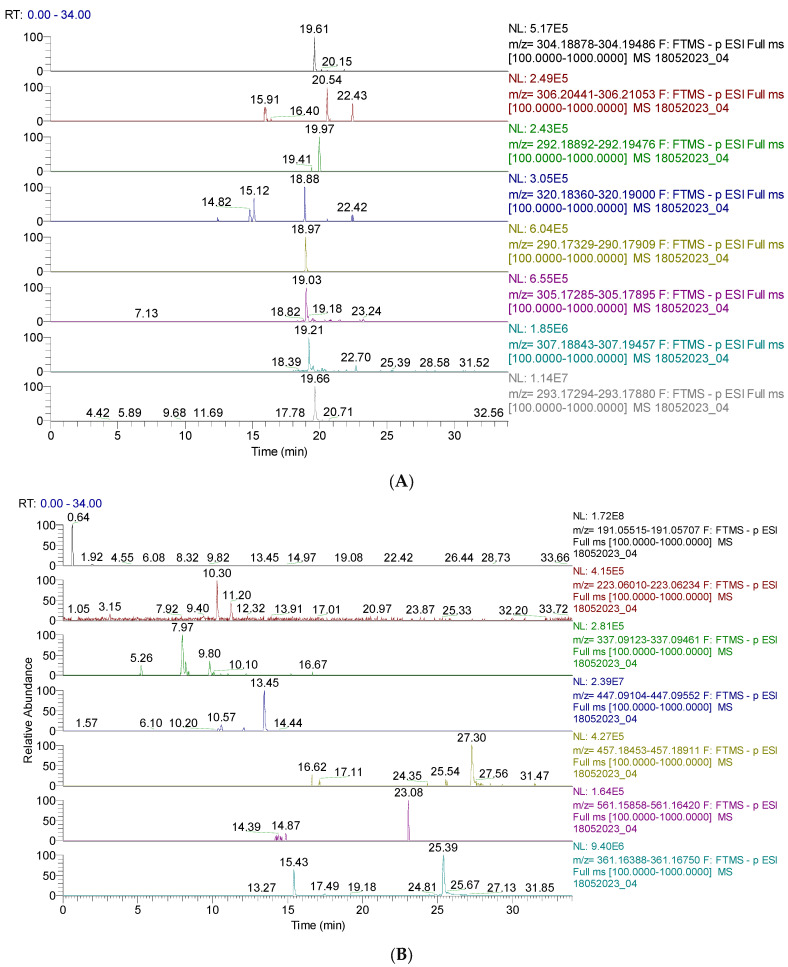
(**A**). UHPLC–HRMS/MS chromatogram of capsaicin derivates identified in CAE; from top to bottom: capsaicin (*m*/*z* = 304.191, Rt = 19.61); dihydrocapsaicin (*m*/*z* = 306.207, Rt = 20.54, 22.43); nordihydrocapsaicin (*m*/*z* = 292.191, Rt = 19.97); capsaicinol (*m*/*z* = 320.186, Rt = 18,88); norcapsaicin (*m*/*z* = 290.176, Rt = 18.97); capsiate (*m*/*z* = 305.175, Rt = 19.03); dihydrocapsiate (*m*/*z* = 307.191, Rt = 19.21); nordihydrocapsiate (*m*/*z* = 293.175, Rt = 19.66); (**B**). UHPLC–HRMS/MS chromatogram of other phenolic compounds identified in CAE; from top to bottom: quinic acid (*m*/*z* = 191.056, Rt = 0.64); sinapic acid (*m*/*z* = 223.061, Rt = 10.30); coumaroylquinic acid (*m*/*z* = 337.092, Rt = 7.27); kaempferol–o–glucoside (*m*/*z* = 447.093, Rt = 13.45); lignan (*m*/*z* = 457.186, Rt = 27.30); lignan P (*m*/*z* = 561.161, Rt = 23.08); secoisolariciresinol (*m*/*z* = 361.165, Rt = 15.43).

The results are shown in Figure 1 and Figure 2 and Table S1 (from the Supplementary Materials). Figure 1 and Figure 2 display the chromatograms of the primary phytochemicals identified in CAE. Figure 1A,B show the main phenolic compounds (flavonoids, isoflavones, and phenolic acids).

Figure 2A,B display the capsaicin derivatives and other phenolic compounds. Capsaicin derivatives are alkaloids with a non-phenolic structure responsible for chili pepper fruits’ specific properties.

The quantified phenolic constituents in CAE are presented in Table 1. 

Table 1 indicates that kaempferol and quercetin have the highest contents (377.26 and 312.02 µg/g), followed by hesperetin (292.81 µg/g), rutin (240.50 µg/g), hyperoside (212.78 µg/g), and chlorogenic acid (207.71 µg/g).

### 3.2. Phenolic Compounds (Polyphenols and Flavonoids) Quantification

The results are presented in Table 2. The analyzed extract has a variable content of secondary metabolites. CAE is richest in total polyphenols with a strong antioxidant effect (4.725 ± 1.361 mg Eq tannic acid/100 g extract). Regarding total flavonoids, CAE shows only 1.154 ± 0.044 mg Eq rutin/100 g extract.

### 3.3. Antioxidant Activity

Significant differences can be observed between the IC_50_/EC_50_ values determined by all three methods (IC_50_DPPH = 1.6699 mg/mL; IC_50_ABTS = 0.2006 mg/mL; EC_50_FRAP = 0.5613 mg/mL, *p* < 0.05), shaping a particular antioxidant profile (Table 2).

### 3.4. CAE-Induced Cytotoxicity

The antiproliferative activity induced by treatments with different CAE concentrations was evaluated in vitro against solid tumor-derived cell lines of different histological origin vs. normal human endothelial cells. Therefore, several CAE-mediated cytotoxicity assays were performed using six adherent tumor cell lines: HEP G2, LoVo, HT-29, MDA-MB-231, SK-OV-3, PE/CA-PJ49, and human umbilical vein endothelial cells (HUVECs), as reference normal cells. 

The cytotoxic activity of CAE was compared to the one induced by several drugs commonly used in oncological treatments (5-fluorouracil (5-FU), cisplatin (CisPt), and doxorubicin (DOX) that were applied throughout all the experiments as positive controls. The concentration range used for 5-fluorouracil (5-FU) and cisplatin (CisPt) was 3.125–200 µM, while for doxorubicin (DOX) the range was between 0.625 and 40 µM (Table 3).

Table 3 shows that 24 h treatments with 5-FU and DOX had almost no influence on HUVEC cell viability for all the concentrations used. The same effect was observed for CisPt concentrations till 100 µM, while for 200 µM of CisPt, a reduction in cell viability to 85.58% was obtained. When treatments of HUVECs were prolonged till 48 h, statistically significant differences in HUVEC cell viability were recorded for all three standard anticancer drugs (CisPt and 5-FU used in higher concentrations than 25 µM; DOX used at more than 10 µM), when compared to exposure for 24 h: 99.36 ± 3.36 vs. 88.63 ± 4.58, *p* < 0.05 (5-FU); 85.58 ± 4.62 vs. 55.80 ± 1.97, *p* < 0.05 (CisPt); and 98.87 ± 5.49 vs. 90.02 ± 0.00, *p* < 0.05 (DOX). 

Treatments with the same ranges of drug concentrations induced higher decreases in cell viabilities, both at 24 and 48 h of exposure, which were time- and dose-dependent and statistically significant (*p* < 0.05, Table 3). The same observation (*p* < 0.05) is available for the tumor cell lines (LoVo, HT-29, HEP G2 for 5-FU; SK-OV-3 and PE/CA-PJ49 for CisPt; and SK-OV-3 and MDA-MB-231 for DOX (Table 3)). Moreover, the cytotoxic effects of oncolytic drugs on tumor cell lines are considerably higher than on HUVECs after 24 and 48 h of exposure (*p* < 0.05, Table 3). 

Further, the CAE antiproliferative capacity was tested on normal human cells and tumor cell lines, as shown in Table 4. 

CAE’s highest concentration (400 µg/mL) decreased the HUVEC cell viability after 24 h of exposure, while lower concentrations of 100 and 200 µg/mL decreased HUVEC viability after exposure for 48 h. The calculated values differ remarkably from the 48 h of contact (99.28 ± 4.86 vs. 83.00 ± 4.44, *p* < 0.05). The 400 µg/mL of CAE significantly decreased the viability of normal cells (90.72 ± 4.86 vs. 52.81 ± 3.59, *p* < 0.05). Between both CAE concentrations (200 and 400 µg/mL), the effects on HUVEC cell viability at 48 h were substantially different (83.00 ± 4.44 vs. 52.81 ± 3.59, *p* < 0.05). At 100 µg/mL, CAE did not affect the normal cell viability after 24 h (Table 4); their prolonged exposure resulted in a low decrease in viability rate (101.91 ± 4.15 vs. 91.27 ± 4.76, *p* > 0.05).

CAE at 100, 200, and 400 µg/mL similarly act on hepatocellular carcinoma HEP G2 (93.95 ± 0.17 vs. 90.75 ± 6.40, *p* > 0.05; 92.61 ± 9.58 vs. 86.00 ± 6.46, *p* > 0.05; 90.65 ± 4.29 vs. 82.02 ± 0.16, *p* < 0.05) and colon adenocarcinoma HT-29 cells (96.38 ± 5.12 vs. 95.35 ± 4.99, *p* > 0.05; 93.04 ± 4.54 vs. 92.05 ± 6.58, *p* > 0.05; 90.40 ± 0.11 vs. 88.08 ± 0.17, *p* > 0.05), slowly reducing their viability. 

On ovarian adenocarcinoma SK-OV-3 cells, CAE of 100, 200, and 400 µg/mL concentrations slowly act after 24 h and considerably decreases tumor cell viability after 48 h (100.15 ± 1.69 vs. 79.08 ± 7.08, *p* < 0.05; 98.01 ± 4.99 vs. 59.93 ± 7.37, *p* < 0.05; 96.36 ± 6.62 vs. 20.32 ± 1.92, *p* < 0.05). Table 4 shows that all viability values after 48 h significantly differed (*p* < 0.05) in the 79.08–20.32% range. 

The same CAE concentrations show notable differences in viability% values on squamous tongue carcinoma PE/CA-PJ49 cells (80.61 ± 3.22 vs. 77.61 ± 3.30, *p* > 0.05; 73.87 ± 3.09 vs. 60.24 ± 7.86, *p* < 0.05; 49.68 ± 1.10 vs. 42.13 ± 2.42, *p* < 0.05). 

They similarly affect the breast adenocarcinoma MDA-MB-231 cells (Table 4) with lower viability values (79.04 ± 5.57 vs. 64.02 ± 7.49, *p* < 0.05; 76.04 ± 0.55 vs. 47.21 ± 7.05, *p* < 0.05; 55.78 ± 5.88 vs. 28.12 ± 5.58, *p* < 0.05). 

The highest decrease in cell viability was registered after 24 and 48 h of CAE contact with LoVo cell line: 71.13 ± 2.10 vs. 60.55 ± 5.74, *p* < 0.05; 56.60 ± 5.38 vs. 30.01 ± 3.16, *p* < 0.05; 43.84 ± 4.70 vs. 8.81 ± 4.49, *p* < 0.05 (Table 4). Moreover, Table 4 indicates that all LoVo cell viability values (at 24 and 48 h) significantly differ. 

The IC_50_ values displayed in Table 4 could be interpreted according to Hidayat et al. [17], resulting in an overview of CAE cytotoxic activity on various tested cell lines. Therefore, CAE shows moderate cytotoxicity—IC_50_ values (µg/mL) < 200 µg/mL—on LoVo and MDA-MB-231 cells after 48 h of exposure, and a low one—IC_50_ = 200–400 µg/mL—on the same cells after 24 h of exposure. On PE/CA-PJ49, CAE exhibits moderate cytotoxic activity after 24 and 48 h; the same effect is recorded on SK-OV-3 after 48 h [17]. Finally, CAE has no cytotoxicity—IC_50_ >> 400 µg/mL—on HEP G2, HT-29, and HUVECs after 24 and 48 h and on SK-OV-3 after 24 h [17]. 

### 3.5. Data Analysis

We illustrated our results of in vitro studies in Figure S1 from the Supplementary Materials for better visualization. Figure S1A,B show a direct proportionality between CAE concentration and cytotoxicity (decrease in cell viability due to the onset of cellular death processes). After 24 h, the highest CAE cytotoxicity is highlighted on LoVo, PE-CA-PJ49, and MDA-MB-231 (Figure S1A). The 48 h treatment shows similar results on HEP G2 and HT-29 (Figure S1B). However, the influence of time contact and concentration is merely evident on other cancer cell lines. The 400 µg/mL CAE concentration had a more intense action, progressively decreasing tumor cell viability in the following order: LoVo, SK-OV-3, PE-CA-PJ49, and MDA-MB-231 (Figure S1B).

#### Principal Component Analysis

**Figure 3:**
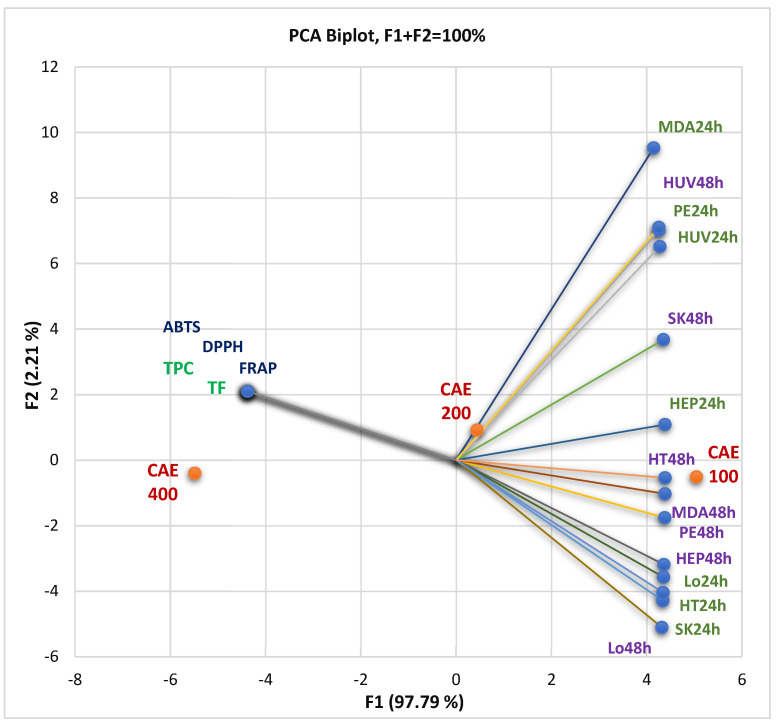
The correlations between TPC and TF, antioxidant effects, and in vitro cytotoxicity of all CAE concentrations (µg/mL). The measured parameter is cell viability% at all three concentrations (100, 200, and 400 µg/mL); CAE = *C. annuum* fruit dry hydro-ethanolic extract; 24 and 48 = the exposition time (hours) of the cell lines on the different CAE concentrations (µg/mL). HUV—HUVECs (endothelial cells); HEP—HEP G2 (human hepatocellular carcinoma); HT—HT-29 (human colon adenocarcinoma); Lo—LoVo (human colon adenocarcinomas); MDA—MDA-MB-231 (human breast adenocarcinoma); PE—PE/CA-PJ49 (human squamous tongue carcinoma); SK—SK-OV-3 (human ovarian adenocarcinoma). TPC—total polyphenols content; TF—total flavonoids.

The correlations between the bioactive phytoconstituents—total phenolic content (TPC) and total flavonoids (TF)—and their pharmacological potential are displayed in Figure 3. Antioxidant activity was assessed by the three methods DPPH, ABTS, and FRAP and expressed as IC_50_ and EC_50_ values (µg/mL), and in vitro cytotoxicity was achieved by MTT assay and defined as cell viability%. At the same time, the PCA biplot from Figure 3 indicates the place of each extract concentration (100, 200, and 300 µg/mL) reported to these correlations. 

Figure 3 shows that TPC and TF significantly correlate with antioxidant activities through all methods (*r* = 0.999, *p* < 0.05). Generally, the TPC, TF, and antioxidant potential negatively correlated with tumor cell viability rate (%) after 24 and 48 h treatment with CAE (Figure 3). That means the cell viability is higher at minimal CAE concentrations because the bioactive secondary metabolites in the low content did not induce significant cellular damage.

Figure 3 and the PCA correlation matrix from the Supplementary Materials show that the viability of SK-OV-3 (ovarian carcinoma), HT-29 (colon adenocarcinoma), and LoVo metastatic cells after 24 h exposure at CAE solutions of different concentrations display a substantial negative correlation with phenolic compounds and antioxidant activity (*r* = −0.998, *r* = −0.997, *r* = −0.999, *p* < 0.05). After 48 h of exposure, the viability percentual value of PE/CA-PJ49 (squamous tongue carcinoma cells), MDA-MB-231 (breast adenocarcinoma), HEP G2 (human hepatocellular carcinoma), and HT-29 cells show the most significant negative correlation (*r* = −0.999, *p* < 0.05) with previously mentioned variable parameters. 

A low cell viability rate highlights a significant process of cellular death; thus, increased antioxidant activity induces a progressive diminution of tumor cell viability associated with the augmentation of cell mortality rate. Therefore, the CAE concentration is directly proportional to the secondary metabolite content (TPC and TF), antioxidant activity, and cytotoxicity. This correlation, more significant in the case of cancer cell lines, could be explained by considering the dual redox behavior of phenolic compounds [18,19,20,21,22]. High concentrations could have pro-oxidant and cytotoxic effects on cancer cell lines, exhibiting, at the same time, an antioxidant and protective action on normal cells [23,24,25,26,27]. Selected references from Table S1 report the pharmacological potential of identified phenolic constituents, focusing on antioxidant and anticancer properties.

In the present study, 400 µg/mL CAE had the highest phenolic and flavonoid content and induced the most substantial cell viability diminution. 

All data suggest that 100 and 200 µg/mL CAE concentrations generally have similar cytotoxicity on tested cell lines, as Figure S2 from Supplementary Material shows, especially after 24 h of exposure. CAE at 400 µg/mL acts significantly differently.

### 3.6. The 48-Hours Acute Toxicity Test Using Daphnia magna and Daphnia Pulex

Capsaicin was tested in low concentrations due to high variability in lethality (L%) within the 7.5–62.5 µg/mL concentration range. 

After 24 h, CAE-induced lethality was L% ≤ 10%. After 48 h, L% = 30–90%, without a precise proportionality concentration–effect (Figure S3 from Supplementary Materials). *D. pulex* exhibited a slightly increased sensitivity than *D. magna;* their responses to the exposure to CAE and capsaicin were similar. CAE had a 48 h LC_50_ < 200 µg/mL, indicating moderate toxicity for both *Daphnia* species; the toxicity was evident after the first 24 h of exposure (Table 5). 

### 3.7. Daphnia Magna Embryonic Development Assay

**Figure 4:**
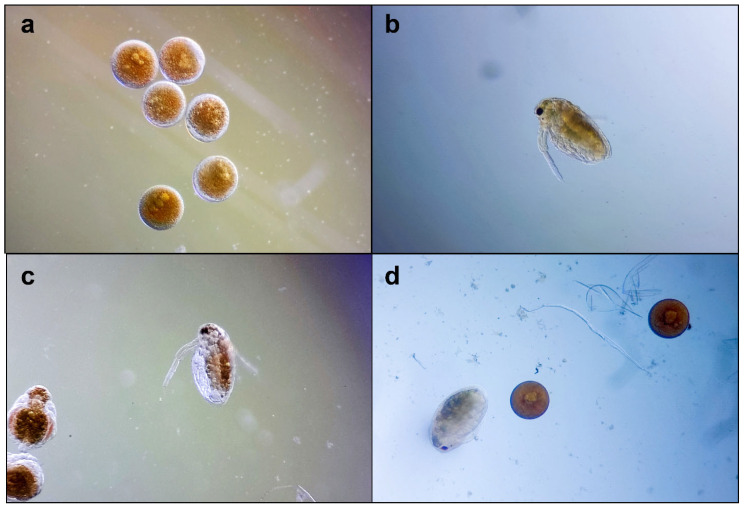
*Daphnia magna* embryonic development assay, microscopic images at 100×: (**a**) embryos before testing; (**b**–**d**) embryos development after 48 h: (**b**) in culture medium; (**c**) in capsaicin 6.25 µg/mL; (**d**) in CAE 31.5 µg/mL.

On the exposed *Daphnia* embryos, a slight to moderate inhibition of general development was observed; a total inhibition was recorded on 3% of the embryos. The retardant effects mainly affected the development of swimming antennae and compound eye formation (Figure 4). These morphological particularities were observed in embryos treated with both samples (CAE and capsaicin); therefore, capsaicin could be considered responsible. 

## 4. Discussion

Chili pepper fruit is considered an indispensable condiment in the current diet in numerous countries due to its health benefits. Chili powder is the most known commercially available product for consumption, and many people purchase *C. annuum* fruit in this form. Therefore, we aimed to buy and investigate *C. annuum* fruit, a commonly manufactured food product accessible and widely consumed by numerous individuals.

We opted for 50% ethanol as a solvent because it is safe and could provide a good extraction yield, being suitable for the solubilization of many phenolic metabolites and other constituents [4]. Reflux extraction is a powerful and highly efficient method. This fact was confirmed in a previous study, where the authors used 60% methanol in water for *C. annuum* fruit powder, comparing it with decoction and microwave-assisted extraction [28]. Our extraction yield was 7.65%. Chilczuk et al. [4] obtained similar values using 80% ethanol for two types of *C. annuum* fruits (6.98% and 7.91%).

Various phenolic phytoconstituents and capsaicin-derived alkaloids were identified in our chili extract by UHPLC–HRMS/MS.

The contents of polyphenols and flavonoids were 472.5 mg Eq tannic acid/g CAE and 115.4 mg Eq rutin/g CAE. In their *C. annuum* fruit extract in 96% ethanol, Bertão et al. [29] reported a TPC = 341.78 mg Eq gallic acid/g and a content of flavonoids of 123.56 mg Eq rutin/g. Chilczuk et al. [4], in their chili extract in 80% ethanol, quantified a TPC = 17.16 and 18.14 mg Eq chlorogenic acid/g and TF = 7.56 and 8.94 mg Eq quercetin/g. Both polyphenol and flavonoid contents were considerably higher in 40% methanol–water fraction: TPC = 40.2 and 51.72 mg Eq chlorogenic acid/g and TF = 41.59 and 61.45 mg Eq quercetin/g. 

Numerous aspects could cause differences. The mode of drying fruits by air, conventional heater, and microwave, the harvesting period, irrigations, and storage time [30] could have a significant influence. However, the main responsible factors for the type and amount of phytocompounds are the solvent used for extraction and the correspondent method. In alcohol–water extracts, the capsaicinoids content is positively correlated with the alcohol concentration because they are practically insoluble in water (https://pubchem.ncbi.nlm.nih.gov/compound/capsaicin#section=solubility, accessed on 1 January 2024). Thus, increasing ethanol concentration from 10% to 75% raises capsaicin solubility by 10–15% [31]. Nine capsaicinoids were identified in CAE through UHPLC–HRMS/MS (Figure 2A and Table S1): capsaicin, dihydrocapsaicin, nordihydrocapsaicin, capsaicinol, norcapsaicin, capsiate, dihydrocapsiate, and nordihydrocapsiate. Capsaicinoids display valuable bioactivities: antioxidant, anti-inflammatory, analgesic, anticancer, and anti-obesity. Hence, they could be helpful in cardiovascular and gastrointestinal diseases, pain relief, weight loss, and cancer prevention and treatment. Other authors obtained *C. annuum* extracts in acetone and hexane, and, using GC/MS analysis, they investigated their composition and reported substantial differences from hydro-ethanolic ones. Besides capsaicinoids, predominant in both extracts, they identified vitamin E, sterols, squalene, aliphatic, and aromatic hydrocarbons, -amyrin (ursolic acid precursor), and small amounts of fatty acids [32].

All phenolic constituents are responsible for CAE antioxidant properties. The DPPH IC_50_ value (1669 µg/mL) evidences a significant capacity of free DPPH radical scavenging. Abdalla et al. [33] reported similar results (DPPH IC_50_ = 1832.25 µg/mL). FRAP EC_50_ of 561 µg/mL is slowly higher than that obtained in the previously mentioned study (451.11 µg/mL). The value of CAE ABTS IC_50_ = 200 µg/mL, highlighting a similar antiradical activity to that recorded by Kim et al. [34] in a previously published study (ABTS IC_50_ = 134.84 µg/mL). Bioactive constituents with a non-phenolic structure can also exhibit an antiradical effect and contribute considerably to pepper extract’s total electron donor capacity [35].

Due to the high systemic toxicity of chemotherapeutics and the progressive increase in multidrug resistance of various cancer cells, the in vitro and in vivo testing of phytochemicals as additional treatments to standard protocols [36] represents a high priority in medical research. Therefore, the plant-based products’ antitumor potential is continuously explored. 

The cytotoxicity of hydro-ethanolic extract of *C. annuum* was evaluated in vitro on the cervical cancer cell line HeLa [37] and colorectal carcinoma (Caco-2 cell line) [38]. All phenolic metabolites quantified in CAE (Table S1) have anticancer properties, mainly due to their pro-oxidant capacity. The anticancer mechanism of capsaicinoids involves NOX-dependent reactive oxygen species suppression, interaction with Ca^2+^-dependent activation of the MAPK pathway, and activation of the p53-mediated mitochondrial apoptosis in cancer cells and other various ones [39]. Due to the most-known anticancer effects, capsaicin is incorporated in multiple pharmaceutical formulations to suppress tumor growth, protect normal tissue against the toxic effects of chemotherapeutic drugs, and increase the classic anticancer drug effects. Table S2 from the Supplementary Materials displays the mechanisms of anticancer effects of capsaicin on various tumor cell lines, as reported in previously published studies. Pure capsaicin and CAE cytotoxicity were investigated in vitro on B104 neuroblastoma cells. This study demonstrated the antitumor effect of capsaicin on the neoplastic cell line. However, CAE did not exhibit a cytotoxic effect on B104 tumor cells. Therefore, co-extracted phenolic compounds in chili pepper ethanol extract could interact antagonistically with the capsaicin cytotoxic action. 

On the other hand, the multi-component chili pepper extracts proved higher cytotoxicity than pure capsaicin on other human cancer cell lines: cervical carcinoma (HeLa), lung carcinoma (A549), breast cancer (MCF-7), and gastric adenocarcinoma (AGS) [40,41,42]. 

In the present study, the cytotoxic activity of CAE was explored on six various tumor cell lines. The results reveal the CAE moderate cytotoxicity [17] (IC_50_ < 200 µg/mL) on LoVo and MDA-MB-231 cells after 48 h of exposure and a low one (IC_50_ = 200–400 µg/mL) on the same cells after 24 h. On PE/CA-PJ49, CAE showed moderate cytotoxic activity (IC_50_ = 200–400 µg/mL) after 24 and 48 h; the same effect was recorded on SK-OV-3 after 48 h. CAE reported low cytotoxicity on SK-OV-3, HEP G2, and HT-29 cells after 24 h (IC_50_ >> 400 µg/mL) [17]. 

Our data also show that CAE has no significant cytotoxicity on HUVEC endothelial cells; similar results were reported in previous studies from the accessed scientific literature. Thus, Chularojmontri et al. [43] found that chili pepper extract and capsaicin improved endothelial function and protected HUVECs against LPS-induced apoptosis. 

A recent study [44] showed the cytotoxic effects of *C. annuum* extracts on various tumor cell lines: breast cancer (MDA-MB-231 and MCF-7), pancreatic cancer (PANC-1, pancreatic ductal adenocarcinoma), skin cancer (A375, human melanoma), and lymphoblastic (K562, myelogenous leukemia). The highest cytotoxicity (IC_50_ = 153.18 µg/mL) was recorded on PANC-1 cells. Similar IC_50_ values were observed on both breast cancer cell lines (IC_50_ = 241.74 and 261.97 µg/mL). The lowest anticancer effects were induced on K562 leukemia and A375 melanoma cells (IC_50_ = 347.67 and 393.58 µg/mL). On normal fibroblasts, it has no cytotoxicity (IC_50_ = 721.28 µg/mL).

On PC3 prostate adenoma and HCT116 human colorectal cancer cell lines, Chilczuk et al. [4] reported substantially higher CAE IC_50_ values (78 mg/mL, 134 mg/mL, respectively). They used L929 murine fibroblasts from subcutaneous adipose tissue as a model of normal cells and obtained a CAE IC_50_ = 90 mg/mL [4]. Recently, another research team incorporated the chili pepper ethanol extract into liposomes and analyzed their bioactivity in the human hepatoma (HEP G2) cell line in vitro [45]. The extract showed no cytotoxic activity and reduced intracellular oxidative stress; the correspondent liposomes with CAE showed improved antioxidant and cytoprotective effects. All previously discussed aspects are similar to our results and show that the cytotoxic activity of chili pepper extract differs on various cell lines.

Using invertebrate species in toxicity assays offers several advantages, per the 3 Rs framework from the bioethical guidelines, and provides simplicity and reproducibility in obtaining toxicological data regarding new compounds or plant extracts [46]. Testing with *Daphnia* sp. is one of the most used approaches in the toxicity assessment of different chemical compounds, including pharmaceuticals [47]. Moreover, using organisms from *Daphnia* species cultured through parthenogenesis ensures significantly reduced variability [48]. Due to high variability in lethality within the 7.5–62.5 µg/mL, we tested capsaicin at lower concentrations (<7.5 µg/mL). Other authors reported that 48 h-LC_50_ = 12.4 µg/mL capsaicin [49]. Moreover, capsaicin can reduce respiratory movements in *Daphnia* sp. [50]. Our in vivo testing results showed moderate CAE cytotoxicity after 48 h (LC_50_ = 178.9 and 148.1 µg/mL), with retardant effects in swimming antennae development and compound eye formation. Similar data about *C. annuum* cytotoxicity were not found in the accessed scientific database. Other authors investigated the cytotoxicity of *C. annuum* extract in 96% ethanol on *A. salina* larvae [29]; they recorded an LC_50_ = 77.98 µg/mL, two times higher than ours. The different animal model susceptibility and higher capsaicinoid content in 96% ethanol extract could explain the results.

## 5. Conclusions

This research investigated a commercially available pre-ground and dried *C. annuum* fruit powder. We obtained a dry extract in 50% ethanol by successive reflux extraction, followed by solvent evaporation and freeze-drying. Through complex UHPLC–HRMS/MS of CAE, we identified 79 bioactive phenolic constituents, including nine capsaicinoids. We also quantified the main classes of phenolic metabolites (polyphenols and flavonoids) and several representatives. The in vitro evaluation of radical scavenging ability and reducing power highlighted the CAE’s significant antioxidant potential. It also displays moderate antiproliferative activity on various tumor cell lines and moderate cytotoxicity and teratogenicity on *Daphnia* sp. A positive correlation exists between exposure time, phenolic metabolite content, and antioxidant and cytotoxic activities.

Our results could enrich the scientific database regarding the composition and bioactivities of various hydro-ethanolic extracts with different ethanol concentrations obtained from *C. annuum* fruits. Generally, our study could be helpful for ordinary people, in the complex analysis of a commercially available product based on chili pepper fruit. 

Further research could investigate the mechanisms implied in the cytotoxic activity of CAE and perform in vivo tests on other animal models. 

## Figures and Tables

**Table 1:** Various phenolic constituents’ content (µg/g) in CAE.

Nr. Crt.	Phenolic Compound	Phytochemical Classification	Content (µg/g)
1	*p*-Coumaric acid	Hydroxycinnamic acid	117.58
2	Chlorogenic acid	Cinnamate ester	207.71
3	Ferulic acid	Hydroxycinnamic acid	24.80
4	Rutin	Flavonoid	240.50
5	Hyperoside	Flavonoid	212.78
6	Naringin	Flavonoid	5.37
7	Genistin	Isoflavone	21.39
8	Hesperetin	Flavonoid	292.81
9	Abscisic acid	Terpenoid	14.65
10	Gallic acid	Hydroxybenzoic acid	60.57
11	Quercetin	Flavonoid	312.02
12	Naringenin	Flavonoid	152.96
13	Kaempferol	Flavanol	377.26
14	Apigenin	Flavonoid	101.31
15	Galangin	Flavonoid	102.10
16	Isorhamnetin	O-methylated flavonol	47.37
17	Chrysin	Flavonoid	19.30
18	Glycitein	Isoflavone	148.91

**Table 2:** Total polyphenols content, total flavonoids, and antioxidant activity of CAE.

Phenolic Compounds		
Total Polyphenols (mg Eq Tannic acid/100 g extract)	Total Flavonoids (mg Eq Rutin/100 g extract)	
4.725 ± 1.361	1.154 ± 0.044	
**Antioxidant Activity**		
IC_50_DPPH (mg/mL)	IC_50_ABTS (mg/mL)	EC_50_FRAP (mg/mL)
1.669	0.200	0.561

DPPH—2,2-diphenyl-1-picryl-hydrazine; ABTS—2,20-azinobis-3-ethylbenzotiazoline-6-sulfonic acid; FRAP—ferric reducing antioxidant power.

**Table 3:** The antiproliferative effects of positive controls on normal cell and tumor cell lines after 24 and 48 h of exposure.

Exposure Time	24 h			
*5-Fluorouracil*				
Cell Line	HUVECs	LoVo	HT-29	HEP G2
**5-FU**	*Cell viability % (mean ± SD)*			
3.125 µM	103.33 ± 7.05	94.26 ± 2.18	96.79 ± 3.59	98.04 ± 6.87
6.25 µM	102.78 ± 4.50	88.04 ± 1.45	92.31 ± 2.01	93.56 ± 0.52
12.5 µM	101.29 ± 2.46	79.31 ± 1.55	84.11 ± 2.69	84.10 ± 4.83
25 µM	103.74 ± 1.39	74.22 ± 3.82	80.87 ± 0.05	78.12 ± 3.61
50 µM	101.54 ± 7.05	62.41 ± 4.00	68.69 ± 4.01	66.85 ± 7.08
100 µM	102.60 ± 4.92	46.00 ± 2.91	54.74 ± 1.42	52.64 ± 3.15
200 µM	99.36 ± 3.36 ^a,b,x^	34.27 ± 2.82 ^a,c,y^	47.40 ± 4.59 ^a,z^	41.69 ± 6.47 ^b,c,w^
IC_50_ (µM)	>200	<100	<200	<200
***Cisplatin***				
**Cell line**	**HUVECs**	**SK-OV-3**		**PE/CA-PJ49**
**CisPt**	*Cell viability % (mean ± SD)*			
3.125 µM	104.65 ± 4.15	99.76 ± 3.65		99.21 ± 1.17
6.25 µM	102.12 ± 4.10	98.44 ± 0.78		97.87 ± 1.51
12.5 µM	101.95 ± 3.69	95.24 ± 2.21		90.77 ± 0.82
25 µM	102.35 ± 3.20	88.20 ± 3.82		86.01 ± 3.02
50 µM	101.10 ± 2.21	79.43 ± 2.50		68.49 ± 4.98
100 µM	100.04 ± 4.08	67.88 ± 0.95		44.95 ± 5.19
200 µM	85.58 ± 4.62 ^a,x^	52.61 ± 4.17 ^a,y^		32.13 ± 4.12 ^a,z^
IC_50_ (µM)	>200	>200		<100
***Doxorubicin***				
**Cell line**	**HUVECs**	**SK-OV-3**		**MDA-MB-231**
**DOX**	*Cell viability % (mean ± SD)*			
0.625 µM	103.67 ± 1.97	98.66 ± 1.91		90.65 ± 2.20
1.25 µM	101.56 ± 2.79	96.06 ± 1.07		77.14 ± 4.67
2.5 µM	102.59 ± 3.20	93.55 ± 1.67		72.99 ± 0.92
5 µM	100.49 ± 5.24	89.00 ± 0.36		62.86 ± 4.78
10 µM	101.74 ± 1.31	82.22 ± 1.91		55.71 ± 1.65
20 µM	100.64 ± 0.41	71.81 ± 2.62		44.81 ± 3.02
40 µM	98.87 ± 5.49 ^a,x^	61.59 ± 1.85 ^a,y^		33.38 ± 6.90 ^a,z^
IC_50_ (µM)	>40	>40		>40
**Exposure Time**	**48 h**			
***5-Fluorouracil***				
**Cell line**	**HUVECs**	**LoVo**	**HT-29**	**HEP G2**
**5-FU**	*Cell viability% (mean ± SD)*			
3.125 µM	105.50 ± 1.72	90.30 ± 4.50	93.08 ± 4.25	96.77 ± 5.75
6.25 µM	103.63 ± 2.04	82.36 ± 2.19	82.16 ± 4.62	90.00 ± 1.79
12.5 µM	102.48 ± 4.96	66.02 ± 5.61	77.37 ± 1.70	81.00 ± 3.47
25 µM	99.02 ± 3.36	59.92 ± 3.98	71.50 ± 5.12	69.12 ± 0.27
50 µM	96.51 ± 2.42	49.30 ± 2.93	55.21 ± 1.25	56.93 ± 4.15
100 µM	93.99 ± 2.29	31.94 ± 3.57	42.78 ± 1.76	46.97 ± 5.44
200 µM	88.63 ± 4.52 ^a,b,x^	13.25 ± 3.49 ^a,b,y^	28.24 ± 7.59 ^a,z^	25.50 ± 6.68 ^b,w^
IC_50_ (µM)	>200	<50	<100	<100
***Cisplatin***				
**Cell line**	**HUVECs**	**SK-OV-3**		**PE/CA-PJ49**
**CisPt**	*Cell viability % (mean ± SD)*			
3.125 µM	101.2 ± 2.35	94.34 ± 4.56		97.08 ± 3.45
6.25 µM	100.68 ± 1.46	82.70 ± 1.88		90.37 ± 4.75
12.5 µM	96.43 ± 5.70	75.19 ± 0.27		84.28 ± 1.60
25 µM	86.59 ± 0.38	67.87 ± 1.72		73.16 ± 4.26
50 µM	78.02 ± 3.12	55.38 ± 6.05		60.26 ± 6.52
100 µM	68.79 ± 1.02	34.32 ± 3.97		33.06 ± 5.19
200 µM	55.80 ± 1.97 ^b,c,x^	29.77 ± 2.04 ^b,y^		21.18 ± 6.53 ^c,z^
IC_50_ (µM)	>200	<100		<100
***Doxorubicin***				
**Cell line**	**HUVECs**	**SK-OV-3**		**MDA-MB-231**
**DOX**	*Cell viability % (mean ± SD)*			
0.625 µM	101.08 ± 0.57	88.91 ± 2.58		85.04 ± 0.95
1.25 µM	103.29 ± 5.41	81.30 ± 2.63		74.76 ± 0.51
2.5 µM	101.15 ± 4.07	77.86 ± 1.13		68.99 ± 3.58
5 µM	100.26 ± 1.78	71.86 ± 4.35		53.84 ± 6.21
10 µM	94.72 ± 0.95	62.91 ± 1.18		46.51 ± 4.30
20 µM	91.97 ± 2.04	55.54 ± 1.07		37.81 ± 6.33
40 µM	90.02 ± 0.00 ^b,x^	36.42 ± 3.43 ^b,y^		19.21 ± 1.91 ^b,z^
IC_50_ (µM)	>40	<40		<10

HUVECs—human umbilical endothelial cells; HEP G2—human hepatocellular carcinoma; HT-29 and LoVo—human colon adenocarcinomas; MDA-MB-231—human breast adenocarcinoma; PE/CA-PJ49—human squamous tongue carcinoma; SK-OV-3—human ovary adenocarcinoma; SD—standard deviation. The superscript letters indicate the significant statistical differences (*p* < 0.05): ^a,b,c^ in the same column, between rows; ^x,y,z,w^ in the same row, between columns. Interpretation of IC_50_ values is based on [16]: IC_50_ ≤ 10 µM = good cytotoxicity, 10 µM < IC_50_ ≤ 30 µM = low cytotoxicity; IC_50_ > 30 µM = inactive. Data shown are expressed as mean values ± standard deviations (SD) of three different experiments (*n* = 3).

**Table 4:** The cytotoxicity of CAE on normal cell and tumor cell lines after 24 and 48 h of exposure.

Exposure Time	24 h			48 h		
*MTS Result*	*Cell Viability%*					
*Mean*/*SD*	*Mean*	*SD*	*IC_50_ (µg/mL)*	*Mean*	*SD*	*IC_50_ (µg/mL)*
** CAE **	** HUVECs **					
6.25 µg/mL	107.78 ^a^	2.47		104.33 ^a^	7.34	
12.5 µg/mL	105.28 ^b^	7.24		101.10 ^b^	5.54	
25 µg/mL	107.22 ^c^	5.33		103.21 ^c^	4.35	
50 µg/mL	103.35	6.74	>>400	97.77 ^d^	3.10	>400
100 µg/mL	101.91	4.15		91.27 ^a,c^	4.76	
200 µg/mL	99.28 ^x^	6.53		83.00 ^b,d,x^	4.44	
400 µg/mL	90.72 ^a,b,c,x^	4.86		52.81 ^a,b,c,d,x^	3.59	
** CAE **	** HEP G2 **					
6.25 µg/mL	99.01	5.59		99.17 ^a^	8.90	
12.5 µg/mL	97.40	9.26		95.16 ^b^	2.66	
25 µg/mL	96.04	0.47		94.20 ^c^	3.58	
50 µg/mL	94.92	0.64	>>400	91.75 ^d^	1.95	>>400
100 µg/mL	93.95	0.17		90.75	6.40	
200 µg/mL	92.61	9.58		86.00	6.46	
400 µg/mL	90.65 ^x^	4.29		82.02 ^a,b,c,d,x^	0.16	
** CAE **	** HT-29 **					
6.25 µg/mL	100.59 ^a,b^	0.11		99.86 ^a^	6.90	
12.5 µg/mL	99.73 ^c^	3.38		97.47 ^b^	5.04	
25 µg/mL	98.75 ^d^	0.37		96.75 ^c^	4.25	
50 µg/mL	97.59	7.18	>>400	96.83	6.40	>>400
100 µg/mL	96.38	5.12		95.35	4.99	
200 µg/mL	93.04 ^a^	4.54		92.05	6.58	
400 µg/mL	90.40 ^b,c,d^	0.11		88.08 ^a,b,c^	0.17	
** CAE **	** LoVo **					
6.25 µg/mL	99.55 ^a,b^	1.61		97.88 ^a,^	8.71	
12.5 µg/mL	97.06 ^c^	4.33		94.24 ^b^	7.44	
25 µg/mL	91.62 ^a^	3.21		89.59 ^c^	3.13	
50 µg/mL	85.13 ^b,c^	3.21	200–400	80.29 ^a,d^	6.30	<200
100 µg/mL	71.13 ^a,b,c,x^	2.10		60.55 ^a,b,c,d,x^	5.74	
200 µg/mL	56.60 ^a,b,c,x^	5.38		30.01 ^a,b,c,d,x^	3.16	
400 µg/mL	43.84 ^a,b,c,x^	4.70		8.81 ^a,b,c,d,x^	4.49	
** CAE **	** MDA-MB-231 **					
6.25 µg/mL	98.77 ^a,b^	5.80		82.96 ^a,b^	5.47	
12.5 µg/mL	96.77 ^c^	4.04		79.12 ^c^	1.03	
25 µg/mL	93.19 ^d^	3.59		77.99 ^d^	6.39	
50 µg/mL	86.95 ^a,c,^	4.22	>400	70.72 ^a^	6.60	<200
100 µg/mL	79.04 ^b,d,x^	5.57		64.02 ^b,c,d,x^	7.49	
200 µg/mL	76.04 ^a,c,d,x^	0.55		47.21 ^a,b,c,d,x^	7.05	
400 µg/mL	55.78 ^a,b,c,d,x^	5.88		28.12 ^a,b,c,d,x^	5.58	
** CAE **	** PE/CA-PJ49 **					
6.25 µg/mL	99.52 ^a,b^	7.61		95.79 ^a^	5.79	
12.5 µg/mL	97.99 ^c^	2.13		92.96 ^b^	4.28	
25 µg/mL	95.64 ^d^	6.93		90.20 ^c^	7.39	
50 µg/mL	91.14	7.68	200–400	86.15 ^d^	5.51	200–400
100 µg/mL	80.61 ^a,c,d^	3.22		77.61 ^a,b,c^	3.30	
200 µg/mL	73.87 ^b,x^	3.09		60.24 ^a,b,c,d,x^	7.86	
400 µg/mL	49.68 ^a,b,c,d,x^	1.10		42.13 ^a,b,c,d,x^	2.42	
** CAE **	** SK-OV-3 **					
6.25 µg/mL	109.96 ^a^	5.65		102.58 ^a^	9.87	
12.5 µg/mL	105.91	6.41		99.18 ^b^	0.73	
25 µg/mL	101.03	4.10		96.09 ^c^	4.02	
50 µg/mL	102.87	1.42	>>400	91.91	5.43	200–400
100 µg/mL	100.15 ^a,x^	1.69		79.08 ^a,b,c,x^	7.08	
200 µg/mL	98.01 ^b,x^	4.99		59.93 ^a,b,c,x^	7.37	
400 µg/mL	96.36 ^c,x^	6.62		20.32 ^a,b,c,x^	1.92	

CAE = *C. annuum* fruit dry hydro-ethanolic extract; 24 and 48 h = cell line exposure time (hours) to the different CAE concentrations (µg/mL). HUVECs—human umbilical endothelial cells; HEP G2—human hepatocellular carcinoma; HT-29 and LoVo—human colon adenocarcinomas; MDA-MB-231 —human breast adenocarcinoma; PE/CA-PJ49—human squamous tongue carcinoma; SK-OV-3—human ovary adenocarcinoma; SD—standard deviation. The superscript letters indicate the significant statistical differences (*p* < 0.05): ^a,b,c,d^ in the same column, between rows; ^x^ in the same row, between columns. Interpretation of IC_50_ values is based on that of the National Cancer Institute [17]: IC_50_ ≤ 20 μg/mL—strong cytotoxic properties, IC_50_ = 21–200 μg/mL—moderate cytotoxicity, IC_50_ = 201–500 μg/mL—low cytotoxicity and IC_50_ ≥ 500 μg/mL—no cytotoxic activity. Data shown are expressed as mean values ± standard deviations (SD) of three different experiments (*n* = 3).

**Table 5:** The results of the 48 h Acute Toxicity Test using *Daphnia* sp.

Time	24 h		48 h	
Parameter	LC_50_ (µg/mL)	95%CI	LC_50_ (µg/mL)	95%CI
* Daphnia magna *				
Capsaicin	NA	NA	NA	NA
CAE	311.0	133.2–726.2	178.9	150.5–212.8
* Daphnia pulex *				
Capsaicin	NA	NA	NA	NA
CAE	261.7	204.7–334.6	148.1	125.4–175.0

CAE—*C. annuum* fruit dry hydro-ethanolic extract; NA— the values could not be calculated, as the maximum L% was 10%.

## Data Availability

Data are available in the manuscript and the Supplementary Materials.

## Supplementary Materials

The following supporting information can be downloaded at https://www.mdpi.com/article/10.3390/pharmaceutics16020245/s1, File S1: Principal Component Analysis; Table S1: Bioactive phenolic constituents and capsaicin-derived compounds identified in CAE by UHPLC HRMS/MS; Table S2. The mechanisms of anticancer effect of capsaicin on various tumor cell lines; Figure S1. The influence of CAE on tumor cell viability at different concentrations after 24 h (A) and 48 h (B) exposure; Figure S2. Agglomerative Hierarchical Clustering (AHC) Dendrogram, where the CAE concentrations (μg/mL) are noted with 100, 200, and 400; Figure S3. The lethality curves were obtained after 24 and 48 h exposure of *Daphnia magna* (a) and *Daphnia pulex* (b) to *Capsicum* extract; error bars represent the SD of two replicates.
