# Supplementary material for: Phytochemical Profile, Antioxidant and Cytotoxic Potential of Capsicum annuum (L.) Dry Hydro-Ethanolic Extract
Source: Pharmaceutics. 2024 Feb 7;16(2):245. doi: 10.3390/pharmaceutics16020245 (PMC10892411; doi:10.3390/pharmaceutics16020245)
Supplement: Supplementary file 1 [file pharmaceutics-16-00245-s001.zip › Tables S1-S2 and Figures S1-S3.pdf]

pharmaceutics

Supplementary Material

Phytochemical Profile, Antioxidant and Cytotoxic Potential of *Capsicum Annuum* (L.) Dry Hydro-Ethanolic Extract

Ionuț Mădălin Ivan[1], Violeta Popovici[2,*], Carmen Lidia Chițescu[3], Liliana Popescu[1], Emanuela Alice Luță[1], Elena Iuliana Ilie[1], Lorelei Irina Brașoveanu[4], Camelia Mia Hotnog[4], Octavian Tudorel Olaru[1*], George Mihai Nițulescu[1], Rica Boscencu[1] and Cerasela Elena Gîrd[1]

[1] Faculty of Pharmacy, University of Medicine and Pharmacy "Carol Davila", Traian Vuia 6, 020956 Bucharest, Romania; ionut.ivan@drd.umfcd.ro (I.M.I.); liliana.popescu22@umfcd.ro (L.P.); emanuela.luta@umfcd.ro (E.A.L.); elena.ionita@drd.umfcd.ro (E.I.I.); octavian.olaru@umfcd.ro (O.T.O.); george.nitulescu@umfcd.ro (G.M.N.); rica.boscencu@umfcd.ro (R.B.); cerasela.gird@umfcd.ro (C.E.G.);
[2] "Costin C. Kirițescu" National Institute of Economic Research—Center for Mountain Economics (INCE-CEMONT), Romanian Academy, 725700 Vatra-Dornei, Romania; violeta.popovici@ce-mont.ro (V.P.)
[3] Faculty of Medicine and Pharmacy, "Dunărea de Jos" University of Galați, A.I. Cuza 35, 800010 Galați, Romania; carmen.chitescu@ugal.ro (C.L.C.);
[4] Center of Immunology, "Stefan S. Nicolau" Institute of Virology, Romanian Academy, 285 Mihai Bravu Ave., 030304 Bucharest, Romania; lorelei.brasoveanu@virology.ro (L.I.B.); camelia.hotnog@virology.ro (C.M.H.);
* Correspondence: violeta.popovici@ce-mont.ro (V.P.); octavian.olaru@umfcd.ro (O.T.O.).

Results

Table S1 displays the bioactive phytochemicals identified in CAE by UHPLC-HRMS/MS, using m/z and Rt values. They belong to different classes of phenolic secondary metabolites (flavonoids, flavanols, flavanones, isoflavones, terpenoids, diterpenes, tannins, phenolic acids, polyphenols, and capsaicin derivatives).

Table S1. Bioactive phenolic constituents and capsaicin-derived compounds identified in CAE by UHPLC HRMS/MS.

Nr. crt.	Identified Compound	Chemical Formula	Adduct Ion/ Monitored Negative Ion	Retention Times (Minutes)	Phytochemical Classification
1	Quercetin	$C_{15}H_{10}O_7$	301.035	14.95	Flavonoid
2	Rutin (Quercetin 3-O-rutinoside)	$C_{27}H_{30}O_{16}$	609.146	13.33	Flavonoid
3	Apigenin	$C_{15}H_{10}O_5$	269.045	16.68	Flavonoid
4	Kaempferol	$C_{15}H_{10}O_6$	285.040	16.49	Flavanol
5	Nepetin (6-methoxy luteolin)	$C_{16}H_{12}O_7$	315.051	16.69	Flavonoid
6	Naringenin	$C_{15}H_{12}O_5$	271.061	15.43	Flavanone
7	Hesperetin	$C_{16}H_{14}O_6$	301.071	15.93	Flavonoid
8	Pinocembrin	$C_{15}H_{12}O_4$	255.066	18.33	Flavonoid
9	Chrysin	$C_{15}H_{10}O_4$	253.050	14.49	Flavonoid
10	Galangin	$C_{15}H_{10}O_5$	269.045	16.68	Flavonoid

11	Hyperoside (Quercetin 3-O-galactoside)	$C_{21}H_{20}O_{12}$	463.087	12.32	Flavonol glicozide
12	Genistin	$C_{21}H_{20}O_{10}$	431.098	13.3	Isoflavone
13	Genistein	$C_{15}H_{10}O_5$	269.045	16.68	Isoflavone
14	Daidzein	$C_{15}H_{10}O_4$	253.050	18.98	Isoflavone
15	Glycitein	$C_{16}H_{12}O_5$	283.061	19.52	Isoflavone
16	Gallic acid	$C_7H_6O_5$	169.014	1.69	Hydroxybenzoic acid
17	Chlorogenic acid	$C_{16}H_{18}O_9$	353.087	6.08	Cinnamate ester
18	Ferulic acid	$C_{10}H_{10}O_4$	193.050	9.92	Hydroxycinnamic acid
19	Abscisic acid	$C_{15}H_{20}O_4$	263.128	14.72	Terpenoid
20	*p*-Coumaric acid	$C_9H_8O_3$	163.039	8.7	Hydroxycinnamic acid
21	Biochanin A	$C_{16}H_{12}O_5$	283.061	19.52	Isoflavone
22	Sissotrin (biochanin A7-O-β-D-glucoside)	$C_{22}H_{22}O_{10}$	445.114	14.03	Flavonoid
23	5,7-Dihydroxy-2'-methoxy Isoflavone	$C_{22}H_{12}O_5$	283.061	19.52	Isoflavone
24	Irilone	$C_{16}H_{10}O_6$	297.040	14.69	Isoflavone
25	Baptigenin	$C_{15}H_{10}O_6$	285.040	15.5	Isoflavone
26	Pratensein/Chrysoeriol	$C_{16}H_{12}O_6$	299.056	16.78/18.39	Isoflavone
27	Irisolidone	$C_{17}H_{14}O_6$	313.071	17.68	Isoflavone
28	Kaempferol-3-O-rutinoside	$C_{27}H_{30}O_{15}$	593.151	9.33	Flavonol glycoside
29	Kaempferol/Luteolin-7-O-β-D-glucoside	$C_{21}H_{20}O_{11}$	447.093	13.45	Flavonoid
30	Chryosoeriol 7-O-glycoside	$C_{22}H_{22}O_{11}$	461.108	14.76	Flavonoid-7-o-glycosides
31	Tricin	$C_{17}H_{14}O_7$	329.066	16.65	Flavonoid
32	Azelaic acid	$C_9H_{16}O_4$	187.097	13.96	Dicarboxylic acid
33	Apigenin 7-O-glucosylglucoside	$C_{27}H_{30}O_{15}$	593.151	9.33	Flavonoid
34	Rosmarinic acid	$C_{18}H_{16}O_8$	359.077	13.41	Ester of caffeic acid
35	Carnosol	$C_{20}H_{26}O_4$	329.175	22.66	Diterpene
36	Carnosic acid	$C_{20}H_{28}O_4$	331.191	21.99	Diterpene
37	Rosmanol/Epirosmanol	$C_{20}H_{26}O_5$	345.170	16.60/19.00	Diterpene
38	Rosmadial/isomers	$C_{20}H_{24}O_5$	343.155	20.35	Diterpene lactone
39	Diosmin (Diosmetin 7-O-rutinoside)	$C_{28}H_{32}O_{15}$	607.166	13.71	Flavonoid
40	Rosmanol methyl ether	$C_{21}H_{28}O_5$	359.186	20.28	Diterpene derivative
41	Neochlorogenic acid	$C_{16}H_{18}O_9$	353.087	6.08	Cinnamate ester
42	Oleanolic acid	$C_{30}H_{48}O_3$	455.353	24.99	Pentacyclic triterpenoid
43	Hispidulin 7-rutinoside/isomers	$C_{28}H_{32}O_{15}$	607.166	13.71	Flavonoid
44	Hispidulin	$C_{16}H_{12}O_6$	299.056	18.39	Flavonoid
45	Salvianolic acid B	$C_{36}H_{30}O_{16}$	717.146	11.72	Polyphenolic acid
46	Gallocatechin/Epigallocatechin	$C_{15}H_{14}O_7$	305.066	1.43	Flavan-3-ol

47	Ursolic acid	$C_{30}H_{48}O_3$	455.353	24.99	Pentacyclic triterpene acid
48	Miquelianin (Quercetin 3-O-glucuronide)	$C_{21}H_{18}O_{13}$	477.067	10.37	Flavonol glucuronide
49	Spicoside A	$C_{30}H_{26}O_{15}$	625.119	13.08	Acylated flavone glycoside
50	Quinic acid	$C_7H_{12}O_6$	191.056	0.64	Cyclohexanecarboxylic acid
51	Coumaroylquinic acid	$C_{16}H_{18}O_8$	337.092	7.97	Quinic acid derivative
52	Quercetin-3-O-rutinoside	$C_{33}H_{40}O_{21}$	771.198	8.93	Flavonoid
53	Sinapic acid	$C_{11}H_{12}O_5$	223.061	10.3	Hydroxycinnamic acid
54	Hidroxy ferulic acid/isomers	$C_{16}H_{20}O_{10}$	371.098	6.63	Hydroxycinnamic acid
55	Valerenic acid	$C_{15}H_{22}O_2$	233.154	21.2	Sesquiterpenoid
56	Quercetin 3-(6-malonyl)-glucoside	$C_{24}H_{22}O_{15}$	549.088	12.82	Flavonoid
57	Lehmannin	$C_{25}H_{28}O_5$	407.186	26.33	Flavanone
58	Alopecurone A	$C_{39}H_{38}O_9$	649.244	16.78	Flavonostilbene
59	Cyanidin 3-O-glucoside	$C_{21}H_{21}ClO_{11}$	483.069	13.45	Anthocyanin
60	Inosine	$C_{10}H_{12}N_4O_5$	267.073	1.38	Nucleoside
61	Cyanidin 3-sambubioside	$C_{26}H_{29}ClO_{15}$	615.112	12.27	Anthocyanidin-3-o-glycoside
62	Taxifolin 3-O-rhamnoside	$C_{21}H_{22}O_{11}$	449.108	12.73	Flavonoid
63	3-Hydroxy-beta-lapachone	$C_{15}H_{14}O_4$	257.081	17.37	o-naphthoquinone
64	Retinoic acid	$C_{20}H_{28}O_2$	299.201	22.38	Retinol derivative
65	Quercetine 3-O-xyloside	$C_{20}H_{18}O_{11}$	433.077	13.31	Flavonoid
66	Lignan	$C_{25}H_{30}O_8$	457.186	27.3	Polyphenolic compound
67	Lignan P	$C_{27}H_{30}O_{13}$	561.161	23.08	Polyphenolic compound
68	Secoisolariciresinol/ isomers	$C_{20}H_{26}O_6$	361.165	15.43/25.39	Polyphenolic compound
69	Vanilic acid glucoside	$C_{14}H_{18}O_9$	329.087	4.4	Hydrolyzable tannin
70	Cyanidin-3-arabinoside	$C_{20}H_{19}ClO_{10}$	453.059	7.17	Anthocyanidin-3-o-glycoside
71	Capsaicin	$C_{18}H_{27}NO_3$	304.191	19.61	Alkaloid
72	Dihydrocapsaicin	$C_{18}H_{29}NO_3$	306.207	20.54/22.43	Alkaloid
73	Nonivamide (Nordihydrocapsaicin)	$C_{17}H_{27}NO_3$	292.191	19.97	Alkaloid
74	Homodihydrocapsaicin	$C_{19}H_{31}NO_3$	320.223	NF	Alkaloid
75	Norcapsaicin	$C_{17}H_{25}NO_3$	290.176	18.97	Alkaloid
76	Capsaicinol	$C_{18}H_{27}NO_4$	320.186	18.88	Alkaloid
77	Capsiate	$C_{18}H_{26}O_4$	305.175	19.03	Alkaloid
78	Dihydrocapsiate	$C_{18}H_{28}O_4$	307.191	19.21	Alkaloid
79	Nordihydrocapsiate	$C_{17}H_{26}O_4$	293.175	19.66	Alkaloid

The results from *in vitro* studies are displayed in Figure S1 for better visualization:

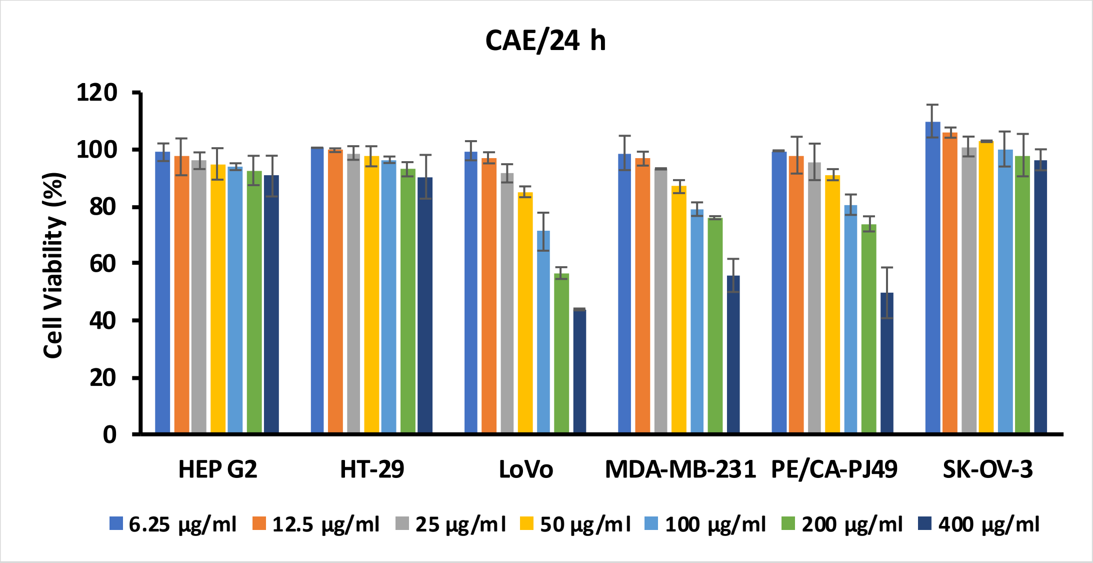

(A)

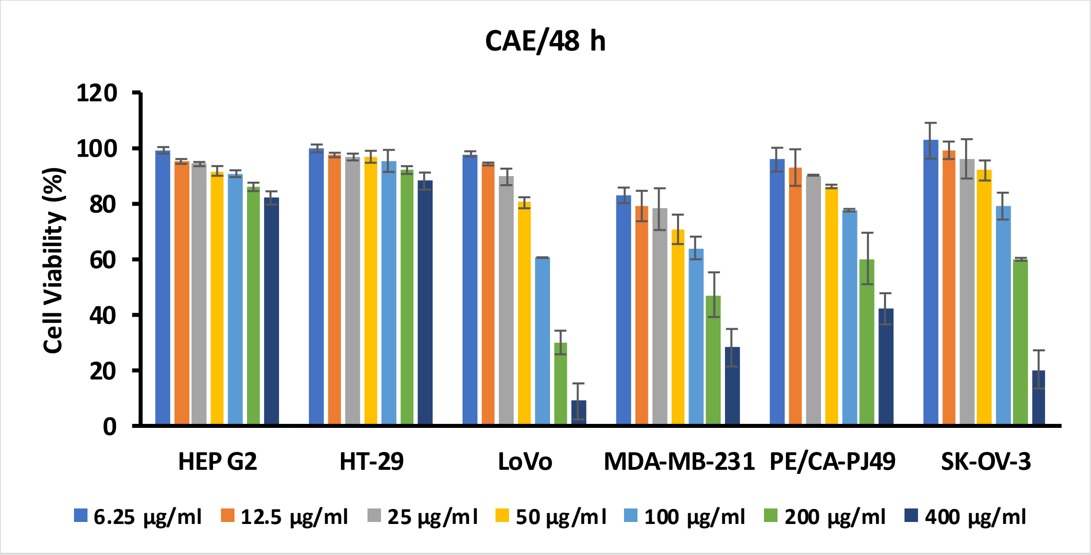

(B)

Figure S1. The influence of CAE on tumor cell viability at different concentrations after 24 hours (A) and 48 hours (B) exposure. CAE = dried *C. annuum* fruits hydro-ethanolic extract, 24 and 48 = the cell lines exposition time (hours) on the different CAE concentrations (µg/mL). HUVEC — human umbilical endothelial cell; HEP G2 — human hepatocellular carcinoma; HT-29 and LoVo — human colon adenocarcinomas; MDA-MB-231 —human breast adenocarcinoma; PE/CA-PJ49 — human squamous tongue carcinoma; SK-OV-3 — human ovary adenocarcinoma; SD — standard deviation. The superscript letters indicate the significant statistical differences ($p < 0.05$): a, b, c, d in the same column, between rows; x in the same row, between columns. Interpretation of IC_{50} values is based on that of the National Cancer Institute [109]: $IC_{50} \leq 20$ µg/mL — strong cytotoxic properties, IC_{50} = 21–200 µg/mL — moderate cytotoxicity, IC_{50} = 201–500 µg/mL — low cytotoxicity, and $IC_{50} \geq 500$ µg/mL — no cytotoxic activity. Data displayed are expressed as mean values ± standard deviations (SD) of three different experiments (n = 3)

Figure S1 (A and B) shows a direct proportionality between CAE concentration and decreased cell line viability. After 24 hours, the highest CAE cytotoxicity is highlighted on LoVo, PE/CA-PJ49, and MDA-MB-231 (Figure S1 A). The 48-hour treatment shows similar results on HEP G2 and HT-29 (Figure S1B). However, the influence of time contact and concentration is merely evident on other cancer cell lines. The 400 µg/mL CAE concentration had a more intense action, progressively decreasing tumor cell viability in the following order: LoVo, SK-OV-3, PE/CA-PJ49, and MDA-MB-231 (Figure S1B).

The results suggest that 100 and 200 µg/mL CAE concentrations generally have similar effects on tested cell line viability, as Figure S2 shows, especially after 24 hours of exposure. CAE at 400 µg/mL acts significantly differently.

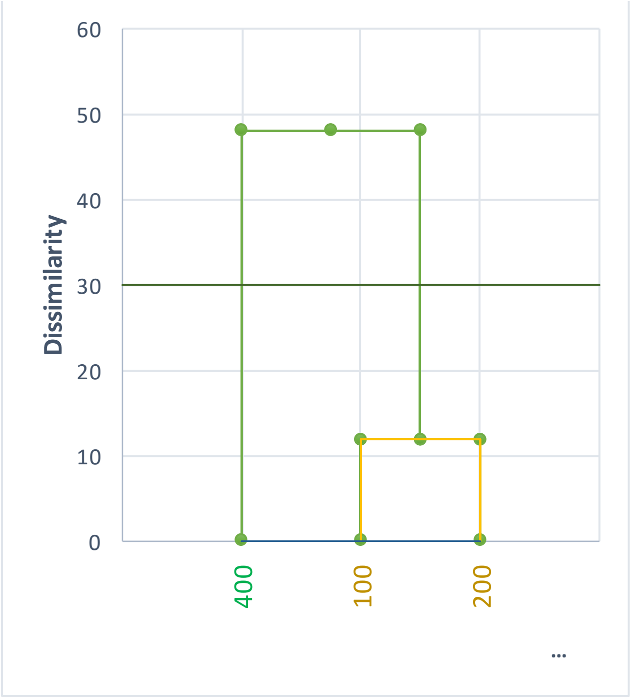

Figure S2. Agglomerative Hierarchical Clustering (AHC) Dendrogram, where the CAE concentrations (µg/mL) are noted with 100, 200, and 400.

48-Hours Acute Toxicity Test Using Daphnia Magna and Daphnia Pulex

Capsaicin was tested in low concentrations due to high variability in lethality (L%) within the 7.5-62.5 µg/mL concentration range.

After 24 hours, CAE-induced lethality was L% ≤ 10%. After 48 hours, L% = 30 - 90%, without a precise proportionality concentration – effect (Figure S3).

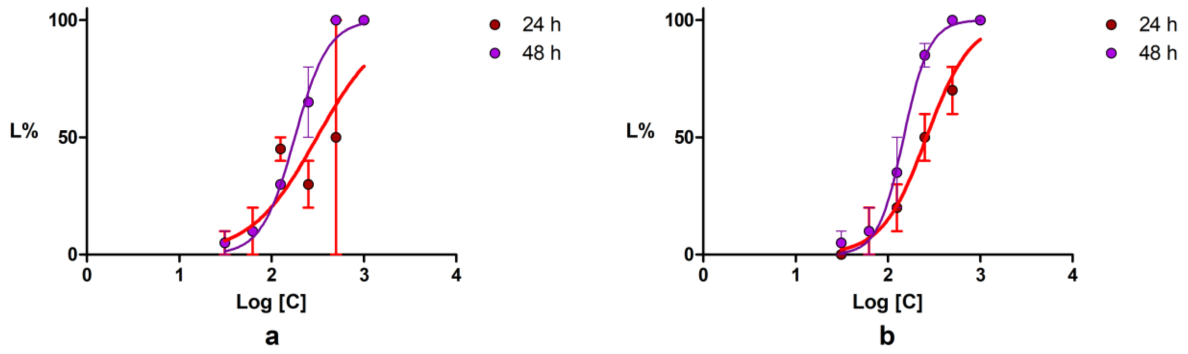

Figure S3. The lethality curves were obtained after 24 and 48 h exposure of *Daphnia magna* (a) and *Daphnia pulex* (b) to *Capsicum* extract; error bars represent the SD of two replicates.

Discussion

Table S2. The mechanisms of anticancer effect of capsaicin on various tumor cell lines

	Cell line	Cancer type	Mechanism of Action
1	MCF-7, BT-20, SK-BR-3, MDA-MB-231, T-47D, BT-474, MCF 10A	Breast cancer	Decreasing of mitochondrial membrane's potential, cell-cycle arrest, apoptosis by down-regulating FBI-1-mediated NF-κB pathway
2	HeLa	Cervical cancer	Premature senescence, apoptosis mitochondrial dysfunction p53 elevation
3	T24 BC 5637 BC A498 RCC	Genito-urinary cancer (Bladder and renal cancer)	Apoptosis Autophagy by AMPK/mTOR-pathway DNA Damage p53/ATM/CHK2 and BRCA1/hTERT mRNA expression Oxidative stress Inhibiting Tumor-Associated NADH Oxidase (tNOX) and Sirtuin1 (SIRT1)
4	U87-MG, U251 U373	Glioblastoma	redox imbalance, ferroptosis, though ACSL4/GPx4 signaling pathways p53 elevation
5	ATC	Thyroid cancer	Autophagy through TRPV1 activation and subsequent calcium influx.
6	HOS MG63	Osteosarcoma	Downregulating SOX2 and EZH2 leads to reduced cancer stemness and

			inhibits metastasis
7	ORL-48	OSCC	Cell death through TRPV1-dependent and -independent pathways. Apoptotic and antiproliferative effect
8	TFK-1, SZ-1	Cholangio-carcinoma	Modulation of Hedgehog pathway, apoptosis
9	SW480, LoVo, HCT 116, CT26, HT-29, COLO 320, COLO205	Colon cancer	Cell cycle arrest, apoptosis, changes in cell morphology, DNA fragmentation
10	AGS, SNU-668, HGC-27	Stomach cancer	Apoptosis, Inhibition of cell proliferation downregulation of several pathways (NADPH, ERK1/2, p38 MAPK, JNK), inhibition of inflammatory molecules (IL-6), increase of apoptotic molecules (caspase-3, p53).
11	AsPC-1, BxPC-3, PANC-1	Pancreatic cancer	Apoptosis by Apoptosis signal-regulating kinase 1-thioredoxin complex dissociation Mediated FOXO-1 Acetylation Inhibition of β-Catenin signaling by disrupting the nuclear β-Catenin/TCF-1 complex
12	Hep G2, Hep 3B	Hepatocellular carcinoma	Oxidative stress and mitochondrial death pathway phosphoinositide 3-kinase/Akt pathway Apoptosis by Involvement of NADPH Oxidase-mediated ROS-Generation
13	LNCaP, PC-3, DU 145	Prostate cancer	Decreasing of mitochondrial membrane's potential, Apoptosis induced by ceramide accumulation, neutral sphingomyelinase, JNK activation
14	A549, H1299, H2009, H23	Lung cancer	AMPK activation through LKB1 Kinase and TRPV1 Receptors suppressing cell growth, inhibition of mitochondrial respiration, increase in intracellular oxygen contents, inhibiting HIF-1α pathway ferroptosis

			by inactivating SLC7A11/GPX4 signaling.
15	CCR-CEM<br>CEM/ADR5000<br>NB4,<br>UF-1,<br>Kasumi-1,<br>HL-60,<br>K-562,<br>KU812<br>U937	Leukemia	Apoptosis,<br>Antiproliferative activity
